# Maternal Exercise Improves High-Fat Diet-Induced Metabolic Abnormalities and Gut Microbiota Profiles in Mouse Dams and Offspring

**DOI:** 10.3389/fcimb.2020.00292

**Published:** 2020-06-17

**Authors:** Liyuan Zhou, Xinhua Xiao, Ming Li, Qian Zhang, Miao Yu, Jia Zheng, Mingqun Deng

**Affiliations:** Key Laboratory of Endocrinology, Department of Endocrinology, Ministry of Health, Translational Medicine Center, Peking Union Medical College, Peking Union Medical College Hospital, Chinese Academy of Medical Sciences, Beijing, China

**Keywords:** gut microbiota, voluntary wheel running, glucose and lipid metabolism, high-fat diet, dams and offspring, intergeneration

## Abstract

Early-life overnutrition programs increased risks of metabolic disorders in adulthood. Regular exercise has been widely accepted to be an effective measure to maintain metabolic health. However, the intergenerational effects of maternal exercise and the specific mechanism are largely unclear. Our objective was to investigate whether maternal exercise could alleviate the metabolic disturbances induced by early-life overnutrition in both dams and offspring and to explore the role of gut microbiota in mediating the effects. C57BL/6 female mice were randomly divided into three groups: the control group, which were fed a normal control diet; high-fat group, which received a high-fat diet; and high-fat with exercise intervention group, which was fed a high-fat diet and received a voluntary wheel running training. The diet intervention started from 3 weeks prior to mating and lasted throughout pregnancy and lactation. The exercise intervention was only prior to and during pregnancy. The male offspring got free access to normal chow diet from weaning to 24 weeks of age. Glucose tolerance test and biochemical parameters were detected in dams at weaning and offspring at 8 and 24 weeks of age. Their cecal contents were collected for the 16 s rDNA amplicon sequencing. The results showed that maternal high-fat diet resulted in significant glucose intolerance, insulin resistance, and lipid profiles disorders in both dams and offspring. Maternal exercise markedly improved insulin sensitivity in dams and metabolic disorders in offspring from young into adulthood. The decrease in unfavorable bacteria and the persistent enrichment of short-chain fatty acids-producers from mothers to adult offspring, particularly the genus *Odoribacter*, were all associated with the improvement of metabolism by maternal exercise. Overall, maternal exercise could significantly mitigate the detrimental effects of a maternal high-fat diet on metabolism in both dams and male offspring. The continuous alterations in gut microbiota might be a critical factor in deciphering the metabolic benefits of maternal exercise, which provides some novel evidence and targets for combating metabolic diseases.

## Introduction

Metabolic disorders, including glucose intolerance, obesity, and hyperlipidemia, have become a global epidemic and brought unprecedented challenges. However, the specific mechanism deciphering the rapid development of metabolic diseases is largely unclear. It is traditionally accepted that genetic susceptibility and environmental factors such as high-calorie diet and physical inactivity in adult life are the major etiology (Gong et al., 2017; Ingelsson and McCarthy, 2018). Over the last few decades, the role of early-life developmental environment in metabolic health gained increasing attention. Substantial evidence has demonstrated that early-life exposures play important roles in determining disease trajectories in later life (Reynolds et al., 2015; Moore, 2017; Prentice, 2018). More specifically, adverse perinatal exposures, including maternal hyperglycemia, hyperlipidemia, and obesity, significantly increased the risk of developing chronic metabolic diseases in later life (Seet et al., 2015; Zambrano et al., 2016; Raghavan et al., 2017; Kawasaki et al., 2018). Therefore, metabolic memory and developmental origins of health and disease (DOHaD) hypothesis have been put forward, which both proposed that developmental environment in early life programmed permanent changes throughout the life (Sata, 2016). Large amounts of studies have confirmed that maternal high-fat diet not only results in metabolic disorders in mothers but elevates the risk of glucose intolerance, insulin resistance, and lipid metabolic disturbances in offspring (Choi, 2018; Keleher et al., 2018). Thus, early life might be a critical window for preventing the intergenerational transmission of metabolic diseases and changing the trajectories of disease development.

The benefits of regular exercise on metabolic diseases have been extensively confirmed (Febbraio, 2017). However, studies exploring the effects of maternal exercise on metabolic health in both mothers and offspring are still limited, especially the clinical trials. In contrast to the deleterious effects of the maternal high-fat diet on metabolism in offspring, several animal studies have demonstrated that maternal exercise before and during pregnancy mitigates the detrimental effects of adverse early-life exposures on glucose tolerance and insulin sensitivity in adult offspring, particularly the male offspring (Stanford et al., 2015; Harris et al., 2018). Regulation of the hepatic gene expression involved in the mitochondrial biogenesis and fatty acid metabolism pathways and alterations in *Pgc1*α methylation in the liver and skeletal muscle of offspring are both considered the potential contributors to the metabolic benefits of maternal exercise (Stanford et al., 2017; Harris et al., 2018). However, the mechanism by which explains the intergenerational metabolic protection of maternal exercise is not completely uncovered. Understanding the mechanistic changes and how they transmit to offspring and persist into adulthood is extremely important to decipher the potential therapeutic benefits of maternal exercise to improve the metabolic health of adult offspring.

More than 1,000 microorganisms are colonizing in the human intestinal tract, which contains 150 times greater genes than that of humans. Therefore, gut microbiota plays crucial roles in host health (Monda et al., 2017). A large amount of evidence has demonstrated that changes in the composition of gut microbiota are associated with multiple diseases, including obesity, diabetes mellitus, and insulin resistance (Gerard, 2016; Munoz-Garach et al., 2016). It has been demonstrated that gut microbiota can be influenced by multiple factors, such as diet, antibiotics, and exercise (Monda et al., 2017). Interestingly, an increasing number of animal models and clinical trials showed that modification of the composition of the gut microbiota might be one of the potential mechanisms for the metabolic benefits of exercise (Monda et al., 2017; Allen et al., 2018a,b). However, studies exploring whether maternal exercise could regulate gut microbiota in both dams and offspring are limited.

In the current study, we aimed to explore the effects of maternal exercise 3 weeks prior to mating and during gestation on metabolic health and to determine whether maternal exercise can mitigate the detrimental effects of a maternal high-fat diet on glucose and lipid metabolism in both dams and offspring. Besides, we investigated the role of gut microbiota in mediating the intergenerational metabolic benefits of maternal exercise and uncovered whether the changes can transmit to offspring and persist into adulthood.

## Materials and Methods

### Animals and Study Design

The C57BL/6 mice were purchased from the Beijing Vital River Laboratory Animal Technology Co., Ltd. (Beijing, China, SCXK-2016-0006). Mice were maintained in a specific pathogen-free (SPF) environment (22 ± 2°C with 12 h light/dark cycle) with *ad libitum* access to food and sterile water during the study. Six-week-old females were randomly divided into three groups after 1 week of acclimation: the control group (maternal control diet group, MC, *n* = 8), which was fed a normal control diet (D12450J) (10% of the calories as fat); high-fat group (maternal high-fat diet group, MHF, *n* = 11), which received a high-fat diet (HFD) (D12492) (60% of the calories as fat); high-fat with exercise intervention group (maternal high-fat diet and exercise group, MHFE, *n* = 8), which was fed a HFD (D12492) and was given a voluntary wheel running training (Yuyan Instruments Co., Ltd, Shanghai, China). The nutritional composition is shown in Supplementary Table S1. Female mice were intervened during 3 weeks before mating and throughout pregnancy. They received their original diets during lactation.

**Figure 1:**
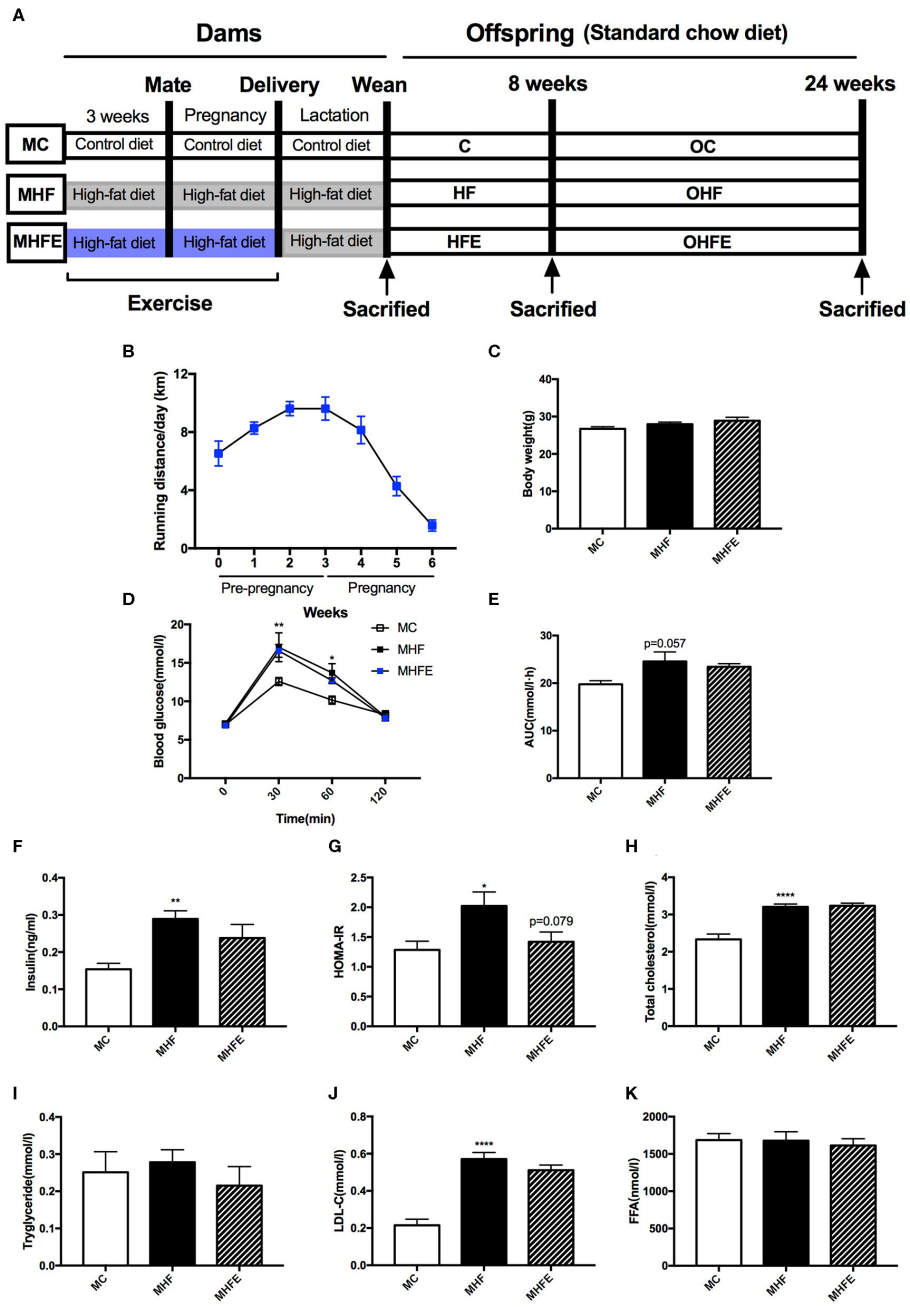
Glucose and lipid metabolism in dams at weaning. **(A)** Experimental scheme; **(B)** Average running distance per day of dams during 3 weeks before mating and throughout pregnancy; **(C)** Body weight in mothers at weaning; **(D)** Maternal blood glucose values following an intraperitoneal glucose tolerance test at weaning; **(E)** AUC of blood glucose values during an intraperitoneal glucose tolerance test; Insulin sensitivity analyses of dams: **(F)** Fasting insulin levels at weaning; and **(G)** HOMA-IR; Maternal serum lipid profiles at weaning: **(H)** TC; **(I)** TG; **(J)** LDL-C; and **(K)** FFA. MC, dams fed the normal control diet; MHF, dams fed the high-fat diet; MHFE, dams intervened with a high-fat diet and exercise. AUC, area under the curve; HOMA-IR, homeostasis model assessment of insulin resistance; TC, total cholesterol; TG, triacylglycerol; LDL-C, low-density lipoprotein cholesterol; FFA, free fatty acid. All of the data are expressed as means ± S.E.M. (MC, *n* = 8; MHF, *n* = 11; MHFE, *n* = 8 in each figure). The statistics were analyzed by one-way ANOVA and two-way ANOVA, with Turkey *post hoc* analyses. Mean values show significant differences between the MC group and the MHF group: ^*^p < 0.05, ^**^p < 0.01, ^****^p < 0.0001.

Female mice were mated with C57BL/6 males on a control diet after the first 3-week intervention. At birth, the litter sizes were culled to 5 pups for each dam to ensure that there is no nutritional bias between litters. Male offspring from the three groups were weaned at postnatal 21 days on a standard chow diet until 24 weeks of age. Running distances of mothers and the body weights of dams and male offspring were measured each week. At weaning, all dams were sacrificed after 10-h fasting for further analyses. At 8 weeks of age, to explore the effects of maternal exercise on metabolic phenotypes and gut microbiota in young offspring, one male per litter of the three groups (offspring from MC at 8 weeks, C, *n* = 6; offspring from MHF at 8 weeks, HF, *n* = 9; offspring from MHFE at 8 weeks, HFE, *n* = 6) were sacrificed. At the end of the experiment (24 weeks of age), one male offspring from different litters (offspring from MC at 24 weeks, OC, *n* = 8; offspring from MHF at 24 weeks, OHF, *n* = 11; offspring from MHFE at 24 weeks, OHFE, *n* = 8) were sacrificed to analyze the long-term effects of maternal exercise on offspring. Blood samples and cecal contents were collected from both mother mice at weaning and one male offspring per litter at 8 and 24 weeks of ages as previously described (Zhou et al., 2019b). The experimental design was shown in Figure 1A. All of the procedures were approved by the animal care and use committee of the Peking Union Medical College Hospital (Beijing, China, SYXC-2014-0029). All of the animal operations were conducted in compliance with the National Institutes of Health guide for the care and use of laboratory animals.

### Intraperitoneal Glucose Tolerance Tests (IPGTT)

The IPGTT was performed in dams at weaning and male offspring at 8 and 24 weeks of age. Mice were fasted for 6 h and were injected intraperitoneally with a glucose load (2 g/kg of body weight). Blood glucose (BG) levels were measured at 0, 30, 60, and 120 min after the injection from tail vein blood using a Contour TS glucometer (Bayer, Beijing, China). The area under the curve (AUC) of the GTT was calculated as previously described (Zheng et al., 2014).

### Serum Biochemical Parameters Measurement

The blood samples collected after 10 h of fasting from dams at weaning and male offspring at 8 and 24 weeks of ages were centrifuged at 3,000 × g for 10 min at 4°C, and the serum was stored in aliquots at −80°C. The serum insulin level was detected using the ELISA kit (80-INSMSU-E01, Salem, NH, USA). Serum total cholesterol (TC), triacylglycerol (TG), low-density lipoprotein cholesterol (LDL-C), and free fatty acids (FFA) were measured as previously described (Zhou et al., 2019c). Insulin sensitivity was assessed using the homeostasis model assessment of insulin resistance (HOMA-IR). The HOMA-IR was calculated as previously described (Zheng et al., 2014).

### Gut Microbiota Analysis

To determine the effects of exercise prior to mating and during pregnancy on maternal and male offspring gut microbiota, we performed the 16 s rDNA amplicon sequencing. Microbial DNA was extracted from the cecal contents of dams at weaning and male offspring at 8 and 24 weeks of ages using a QIAamp DNA Stool Mini Kit (Qiagen, Hilden, Germany) according to manufacturer's protocols. The V3-V4 regions of the 16S rRNA genes were amplified using the primers 341F 5′-CCTACGGGRSGCAGCAG-3′and 806R, 5′-GGACTACVVGGGTATCT AATC-3′, which included barcode sequences and adapters. Amplicons were purified using the AxyPrep DNA Gel Extraction Kit (Axygen Biosciences, Union City, CA, U.S.) and were quantified using Qubit^®^ 2.0 (Invitrogen, U.S.). The tags were sequenced on the Illumina HiSeq 2500 PE250 platform (Illumina, Inc., CA, USA).

A total number of 2,695,371 reads was generated with an average length of 413 bp. The reads were merged by Pandaseq (Masella et al., 2012). After merging paired-end reads, reads were performed by quality filtering, as detailed in the follows:(1) remove the reads with an average quality value below 20; (2) remove the reads with more than 3 N; and (3) the length of the reads is between 220 and 500 nt. High-quality reads were clustered into operational taxonomic units (OTUs) with the 97% similarity using UPARSE software (version 7.0.1001) (Edgar, 2013), and representative sequences for each OTU were screened using QIIME software (version 1.7.0, Quantitative Insights into Microbial Ecology) (Caporaso et al., 2010). Then, the RDP (Release 11.5) (Cole et al., 2014) was used to annotate taxonomic information. Alpha and beta diversity analyses were achieved by QIIME software (Version 1.7.0) and R software (Version 2.15.3). For alpha diversity, Chao1, observed_species, and PD_whole_tree were analyzed. For beta diversity, Principal coordinates analysis (PCoA) plots and Analysis of similarities (ANOSIM) were performed using unweighted UniFrac. Additionally, linear discriminant analysis (LDA) of the effect size (LEfSe) was used to determine differences among the groups.

### Statistical Analysis

The data were expressed as mean ± standard error of the mean (S.E.M). The statistics were analyzed by one-way ANOVA and two-way ANOVA, with Turkey *post hoc* analyses. Correlation analyses between the relative abundance of bacterial taxa at genus levels and metabolic parameters were performed by Spearman correlation coefficient test. A *p*-value <0.05 was considered statistically significant. Prism version 7.0 (GraphPad Software Inc., San Diego, CA, USA) was used for statistical analysis.

## Results

### The Effects of Exercise Prior to Mating and During Pregnancy on Body Weight and Biochemical Parameters in Dams at Weaning

To determine the intergenerational effects of exercise, female mice did a voluntary wheel running training for 3 weeks before mating and throughout pregnancy on a high-fat diet (HFD). As shown in Figure 1B, the average running distance per day was between 6.5 and 9.7 km before mating. They gradually reduced physical activity after mating to nearly 1.5 km each day during the last week of pregnancy. 9-week diet intervention and 6 weeks' exercise training did not affect the body weight in dams at weaning (Figure 1C). To investigate whether a HFD and exercise impacted glucose metabolism in dams, we performed the intraperitoneal glucose tolerance tests (IPGTT) at weaning. Maternal 9 weeks of HFD feeding led to significantly higher blood glucose (BG) levels at 30 min (*p* < 0.01) and 60 min (*p* < 0.05), as well as a trend toward the larger area under the curve (AUC) values (*p* = 0.057) than those of dams in the maternal control diet group (MC) (Figures 1D,E). However, exercise 3 weeks before and during pregnancy did not improve glucose intolerance induced by HFD in dams at weaning. Then we assessed the fasting insulin levels and insulin sensitivity. The results showed that maternal HFD intake significantly increased the serum insulin levels (*p* < 0.01) and the homeostasis model assessment of insulin resistance (HOMA-IR) index (*p* < 0.05) in the mothers *per se* compared with those fed the control diet. Maternal exercise tended to decrease the HOMA-IR index elevated by HFD in dams (*p* = 0.079) (Figures 1F,G).

Additionally, the effects of a HFD and exercise on serum lipid profiles were also evaluated. The serum levels of total cholesterol (TC) (*p* < 0.0001) and low-density lipoprotein cholesterol (LDL-C) (*p* < 0.0001) in HFD-fed dams were higher than those fed the control diet. However, no significant difference was observed in the lipid profiles between the dams in the maternal high-fat diet and exercise group (MHFE) and in the maternal high-fat diet group (MHF) (Figures 1H–K).

### The Changes of Gut Microbiota in Dams at Weaning

**Figure 2:**
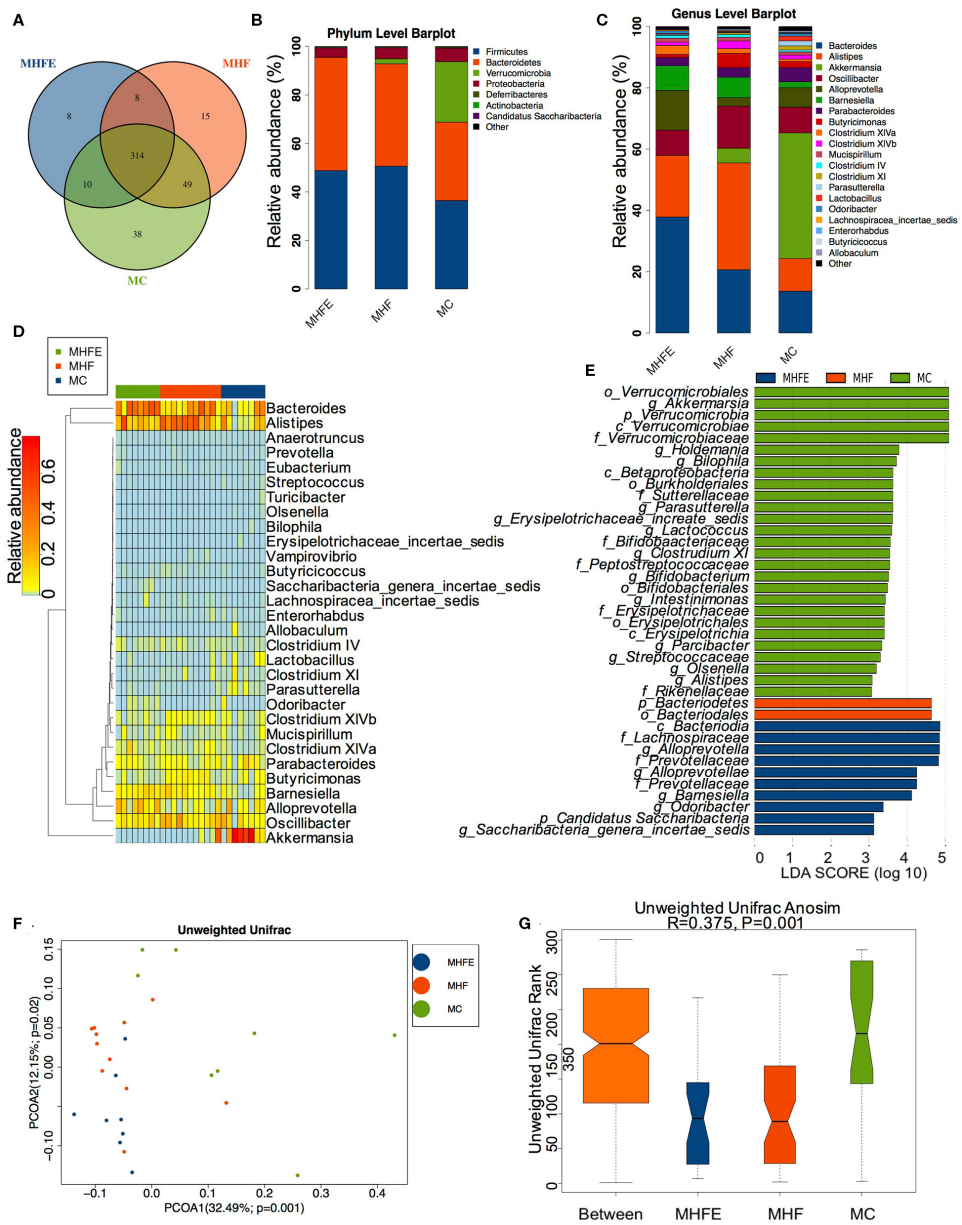
The alterations of gut microbiome in dams at weaning (MC, *n* = 8; MHF, *n* = 11; MHFE, *n* = 8 in each figure). **(A)** Venn diagram of the OUTs; **(B)** Relative abundance of the top 10 phyla; **(C)** Relative abundance of the top 20 species at the genus level; Different species analysis among the three groups: **(D)** Heatmap analysis of the different germs at the genus level; and **(E)** LEfSe analysis of the different intestinal microbiome from the phylum level to the genus level; Beta diversity analysis of gut microbiome: **(F)** PCoA plots of gut microbiome; and **(G)** ANOSIM analysis. MC, dams fed the normal control diet; MHF, dams fed the high-fat diet; MHFE, dams intervened with a high-fat diet and exercise.

To explore the impact of HFD and exercise on gut microbiota in mothers at weaning, we assessed the gut microbiome of cecal contents using the 16 s rDNA amplicon sequencing. The sequencing data have been submitted to the Sequence Read Archive (SRA) database (PRJNA573981). Firstly, we analyzed the shared and unique germs among the three groups by analyzing the operational taxonomic units (OTUs). The results showed that there were 314 shared OTUs, 38 unique OTUs in control diet-fed dams, 15 unique OTUs in HFD-fed dams, and 8 unique OTUs in dams of the exercise intervention group (Figure 2A). Figures 2B,C showed the top 10 phyla and the top 20 species at the genus level, respectively. Firmicutes, Bacteroidetes, Verrucomicrobiota and Proteobacteria were the most abundant germs at the phylum level. The heatmap listed the significantly different genera among the three groups (Figure 2D). To this end, we further dissected the individual microbial species differentially enriched among the three groups from the phylum level to the genus level (Figure 2E). At the genus level, maternal HFD feeding significantly enriched *Alistipes*. it has been reported that the relative abundance of genus *Alistipes* is significantly increased in obese mice (Clarke et al., 2013) and is associated with type 2 diabetes mellitus and other health risks (Rautio et al., 2003; Saulnier et al., 2011; Xu et al., 2015). However, *Parasutterella, Lactococcus, Bifidobacterium*, and *Intestinimonas* were abundant in control diet-fed dams. *Parasutterella* was an important bacteria participating in the maintenance of bile acid and cholesterol metabolism, which is a member of the healthy intestinal microbiota in human gastrointestinal tract. Previous studies demonstrated that a significant decrease in the abundance of *Parasutterella* was associated with various diseases, including obesity, diabetes, and non-alcohol fatty liver disease (Willing et al., 2010; Ju et al., 2019), whereas the metabolic improvement induced by diet intervention was associated with the significant enrichment of *Parasutterella* (Cheng et al., 2018). *Bifidobacterium* has been shown to play a crucial role in host health and has multiple functions, including production of antioxidants, maintenance of immune homeostasis, and protection of gut barrier (Riviere et al., 2016). *Intestinimonas* was shown to have beneficial effects on health as an important butyrate producer (Klaring et al., 2013; Bui et al., 2015). Exercise dramatically increased the abundance of *Alloprevotella, Barnesiella, Odoricbacter*, and *Saccharibacteria_genera_incertae_sedis*. Furthermore, the assessment of alpha diversity showed no significant differences in richness and diversity of microbiota among the three groups (Supplementary Figure S1). However, the intestinal microbial communities were well-separated in dams among the three groups (ANOSIM R = 0.375, *p* = 0.001) (Figures 2F,G).

### The Impact of Maternal Exercise on Glucose and Lipid Metabolism in Male Offspring at 8 Weeks of Age

**Figure 3:**
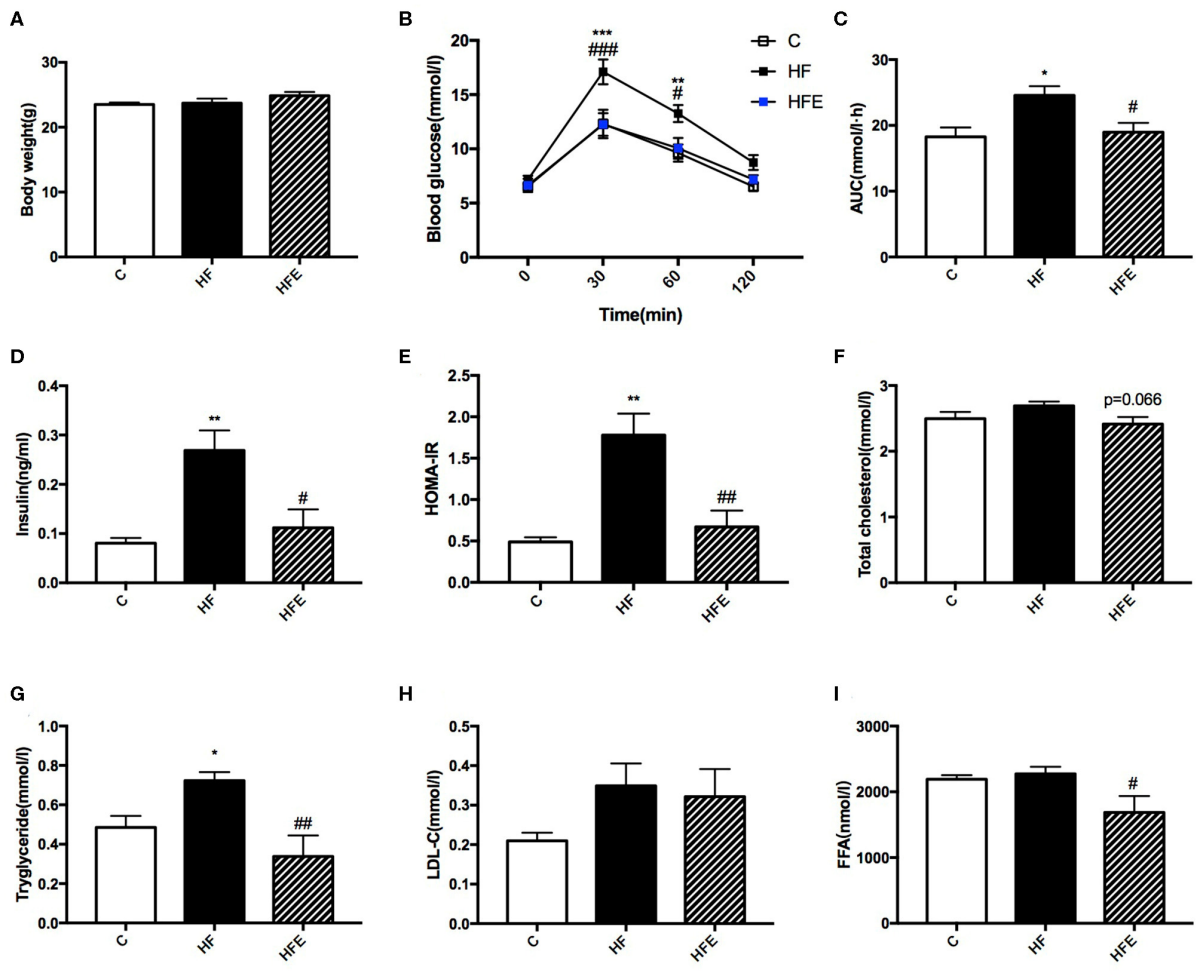
Metabolic parameters in male offspring at 8 weeks of age. **(A)** Offspring body weight; **(B)** Blood glucose values following an intraperitoneal glucose tolerance test; **(C)** AUC of blood glucose levels during glucose tolerance test; Insulin sensitivity analyses of young offspring: **(D)** Fasting serum insulin levels; and **(E)** HOMA-IR; Fasting serum lipid profiles analysis in offspring at 8 weeks of age: **(F)** TC; **(G)** TG; **(H)** LDL-C; and **(I)** FFA. C, offspring of dams fed the normal control diet fed; HF, offspring of dams fed the high-fat diet; HFE, offspring of dams intervened with a high-fat diet and exercise. AUC, area under the curve; HOMA-IR, homeostasis model assessment of insulin resistance; TC, total cholesterol; TG, triacylglycerol; LDL-C, low-density lipoprotein cholesterol; FFA, free fatty acid. Data are expressed as means ± S.E.M. (C, *n* = 6; HF, *n* = 9; HFE, *n* = 6 in each figure). The statistics were analyzed by one-way ANOVA and two-way ANOVA, with Turkey *post hoc* analyses. Mean values show significant differences between C group and the HF group: ^*^p < 0.05, ^**^p < 0.01, ^***^p < 0.001; Mean values show significant differences between HF group and the HFE group during: ^#^*p* < 0.05, ^##^*p* < 0.01, ^###^*p* < 0.001.

Besides the phenotype in mothers, we further investigated the intergenerational influence of maternal HFD and exercise on glucose and lipid metabolism in male offspring at 8 weeks of age. Consistent with dams, body weight among the three groups was not significantly different (Figure 3A). In terms of the glucose tolerance, maternal HFD intake dramatically elevated the BG levels at 30 min (*p* < 0.001) and 60 min (*p* < 0.01) during the IPGTT and led to larger AUC (*p* < 0.05) in offspring compared with those of offspring form control diet-fed dams. By contrast, maternal exercise significantly reduced the BG levels (*p* < 0.001 and *p* < 0.05) and the AUC (*p* < 0.05) induced by maternal HFD (Figures 3B,C). The serum insulin levels and HOMA-IR index were also assessed in male offspring. The results showed that 9 weeks of maternal HFD significantly increased the insulin levels (*p* < 0.01) and HOMA-IR index *(p* < 0.01) in male offspring at 8 weeks of age compared with those of offspring from control diet-fed dams, which were dramatically decreased by maternal exercise (*p* < 0.05 and *p* < 0.01) (Figures 3D,E). As for the lipid metabolism, maternal HFD feeding also significantly elevated the TC levels (*p* < 0.05) in young male offspring compared with those of offspring from MC group (C), whereas maternal exercise obviously reduced the serum TG (*p* < 0.01) and FFA (*p* < 0.05) levels elevated by maternal HFD (Figures 3F–I).

### The Alterations of Intestinal Microbial Community in Male Offspring at 8 Weeks of Age

**Figure 4:**
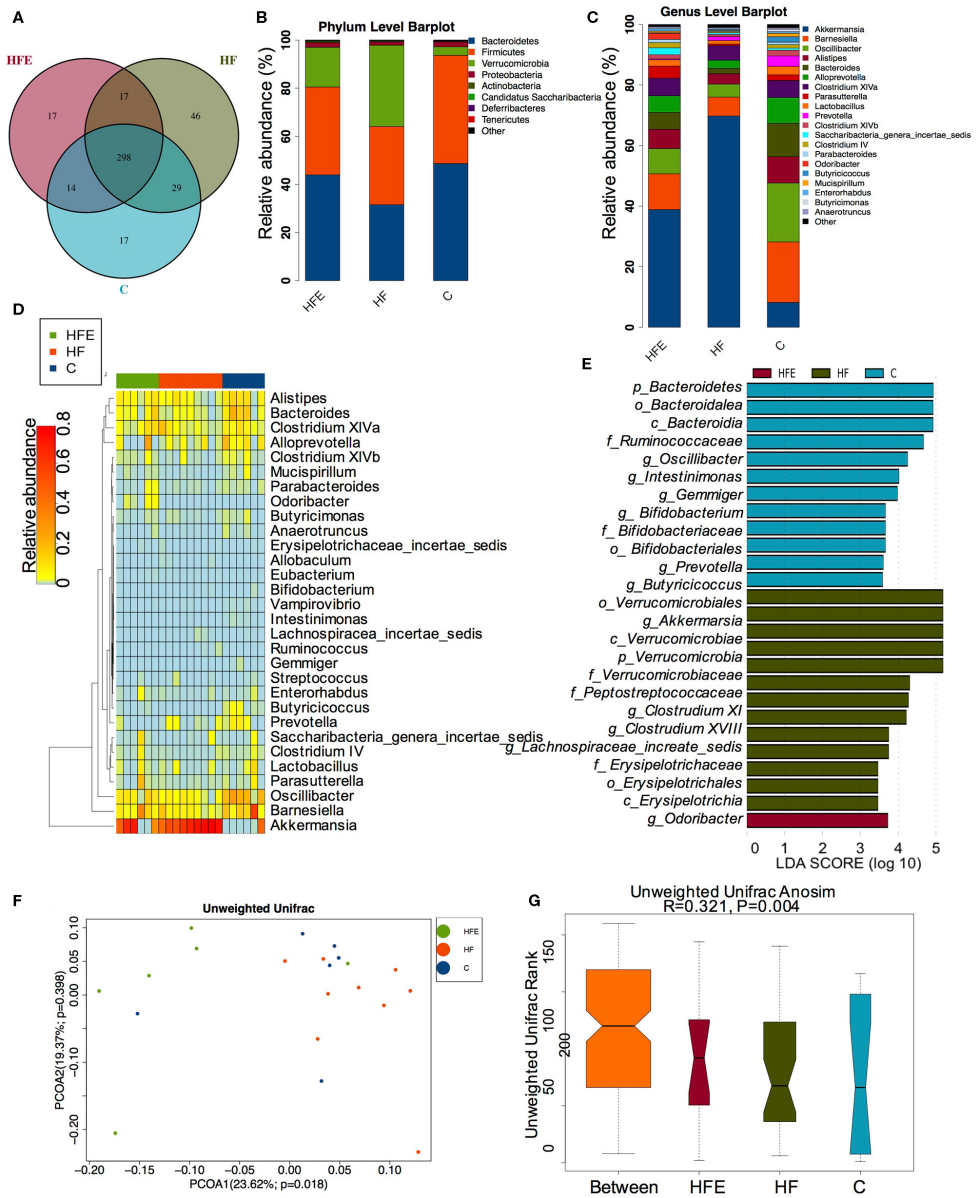
The changes of gut microbiome in male offspring at 8 weeks of age (C, *n* = 6; HF, *n* = 9; HFE, *n* = 6 in each figure). **(A)** Venn diagram of the OUTs; **(B)** Relative abundance of the top 10 species at the phylum level; **(C)** Relative abundance of the top 20 germs at the genus level; Different species analysis among the three groups: **(D)** Heatmap analysis of the different species at the genus level; **(E)** LEfSe analysis of the significantly enriched gut microbiome from the phylum level to the genus level; Beta diversity analysis of gut microbiome: **(F)** PCoA plots of gut microbiome; and **(G)** ANOSIM analysis. C, offspring of dams fed the normal control diet fed; HF, offspring of dams fed the high-fat diet; HFE, offspring of dams intervened with a high-fat diet and exercise.

Given maternal intervention impacted metabolic phenotype in young offspring, we explored the effects of maternal HFD and exercise on gut microbiota in male offspring at 8 weeks of age. 298 shared OTUs, 17 unique OTUs in offspring of the control diet-fed dams, 46 unique OTUs in offspring of the HFD-fed dams, and 17 unique OTUs in the offspring from the MHF group (HFE) were shown in the Venn diagram (Figure 4A). Consistent with the composition of microbiota in dams at weaning, Firmicutes, Bacteroidetes, Verrucomicrobiotam, and Proteobacteria were also the most abundant species at the phylum level (Figure 4B). Top 20 genera were listed in Figure 4C and markedly different species at the genus level in male offspring at 8 weeks of age among the three groups were concluded in the heatmap (Figure 4D). At the genus level, maternal HFD feeding significantly enriched *Lachnospiracea_incertae_sedis* and *Allobaculum* in male offspring. The relationships between the abundance of *Allobaculum* and metabolic dysbiosis, dietary fat intake, and lipid profiles have been reported in several studies (Cani et al., 2008; Ravussin et al., 2012; Nobel et al., 2015). Zhou et al. further confirmed that the enriched *Allobaculum* might play important roles in the procession of diabetes in rats (Zhou et al., 2019d). *Intestinimonas, Bifidobacterium*, and *Butyricicoccus* were abundant in the offspring of the control diet-fed dams. In addition to *Intestinimonas, Butyricicoccus* was also a butyrate-producing bacteria. *Butyricicoccus* is a member of the family *Ruminococcaceae* from *clostridial cluster* IV and can protect the gut barrier (Devriese et al., 2017; Trachsel et al., 2018). Clinical trials and experimental animal models all reported that the metabolic improvement of multiple diet intervention was associated with the increased abundance of *Butyricicoccus* (Pataky et al., 2016; Suriano et al., 2017; Gonzalez-Sarrias et al., 2018). Maternal exercise dramatically increased the abundance of *Odoricbacter* in the offspring of the HFE group (Figure 4E). No significant differences in alpha diversity were observed among the three groups (Supplementary Figure S2). But the gut microbiota was widely separated among the three groups (Figure 4F). ANOSIM analysis supported that the differences were significant (*R* = 0.321, *p* = 0.001) (Figure 4G).

### The Effects of Maternal Exercise on Biochemical Parameters in Male Offspring at 24 Weeks of Age

**Figure 5:**
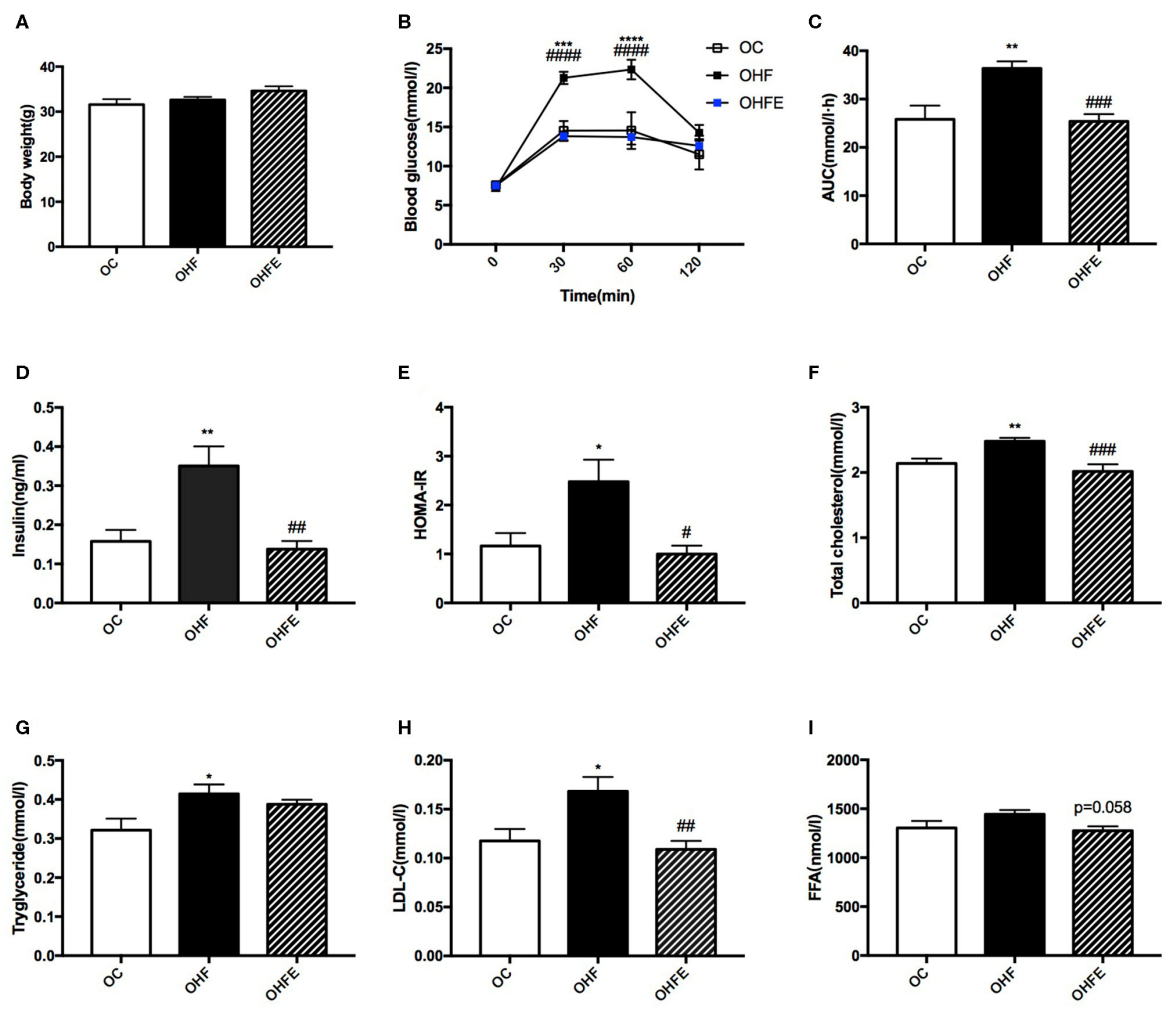
Metabolic parameters in male offspring at 24 weeks of age. **(A)** Body weight; **(B)** Blood glucose values following an intraperitoneal glucose tolerance test; **(C)** AUC of blood glucose levels during glucose tolerance test; Insulin sensitivity analyses of offspring at 24 weeks: **(D)** Fasting serum insulin levels; and **(E)** HOMA-IR; Fasting serum lipid profiles analysis in offspring at 24 weeks of age: **(F)** TC; **(G)** TG; **(H)** LDL-C; and **(I)** FFA. OC, offspring of dams fed the normal control diet; OHF, offspring of dams fed the high-fat diet; OHFE, offspring of dams intervened with a high-fat diet and exercise. AUC, area under the curve; HOMA-IR, homeostasis model assessment of insulin resistance; TC, total cholesterol; TG, triacylglycerol; LDL-C, low-density lipoprotein cholesterol; FFA, free fatty acid. Data are expressed as means ± S.E.M. (OC, *n* = 8; OHF, *n* = 11; OHFE, *n* = 8 in each figure). The statistics were analyzed by one-way ANOVA and two-way ANOVA, with Turkey *post hoc* analyses. Mean values show significant differences between OC group and the OHF group: ^*^p < 0.05, ^**^p < 0.01, ^***^p < 0.001, ^****^p < 0.0001; Mean values show significant differences between OHF group and the OHFE group: ^#^*p* < 0.05, ^##^*p* < 0.01, ^###^*p* < 0.001, ^####^*p* < 0.0001.

To detect whether the changes of metabolism in offspring were persistent during aging, the glucose and lipid metabolic parameters in male offspring at 24 weeks of age were further investigated. As shown in Figure 5A, no significant difference in body weight among the three groups was observed. However, the BG levels at 30 min (*p* < 0.001) and 60 min (*p* < 0.0001) during the IPGTT and the AUC (*p* < 0.01) in offspring were all higher than those in offspring of the control diet-fed dams. Maternal exercise also significantly normalized the BG levels (*p* < 0.0001 and *p* < 0.0001) and the AUC (*p* < 0.001) elevated by maternal HFD (Figures 5B,C). Moreover, the insulin levels (*p* < 0.01) and HOMA-IR index (*p* < 0.05) in male offspring at 24 weeks of age were also significantly increased compared with those of offspring from MC group (OC), which were almost normalized by maternal exercise (*p* < 0.01 and *p* < 0.05) (Figures 5D,E). In terms of the lipid metabolism, maternal HFD significantly elevated the TC (*p* < 0.01), TG (*p* < 0.05) and LDL-C levels (*p* < 0.05) in male offspring compared with those of offspring in the OC group, whereas maternal exercise improved the serum TC (*p* < 0.001), LDL-C (*p* < 0.01) and seemed to decrease FFA (*p* = 0.058) levels (Figures 5F–I).

### The Changes of Gut Microbiota in Adult Male Offspring

**Figure 6:**
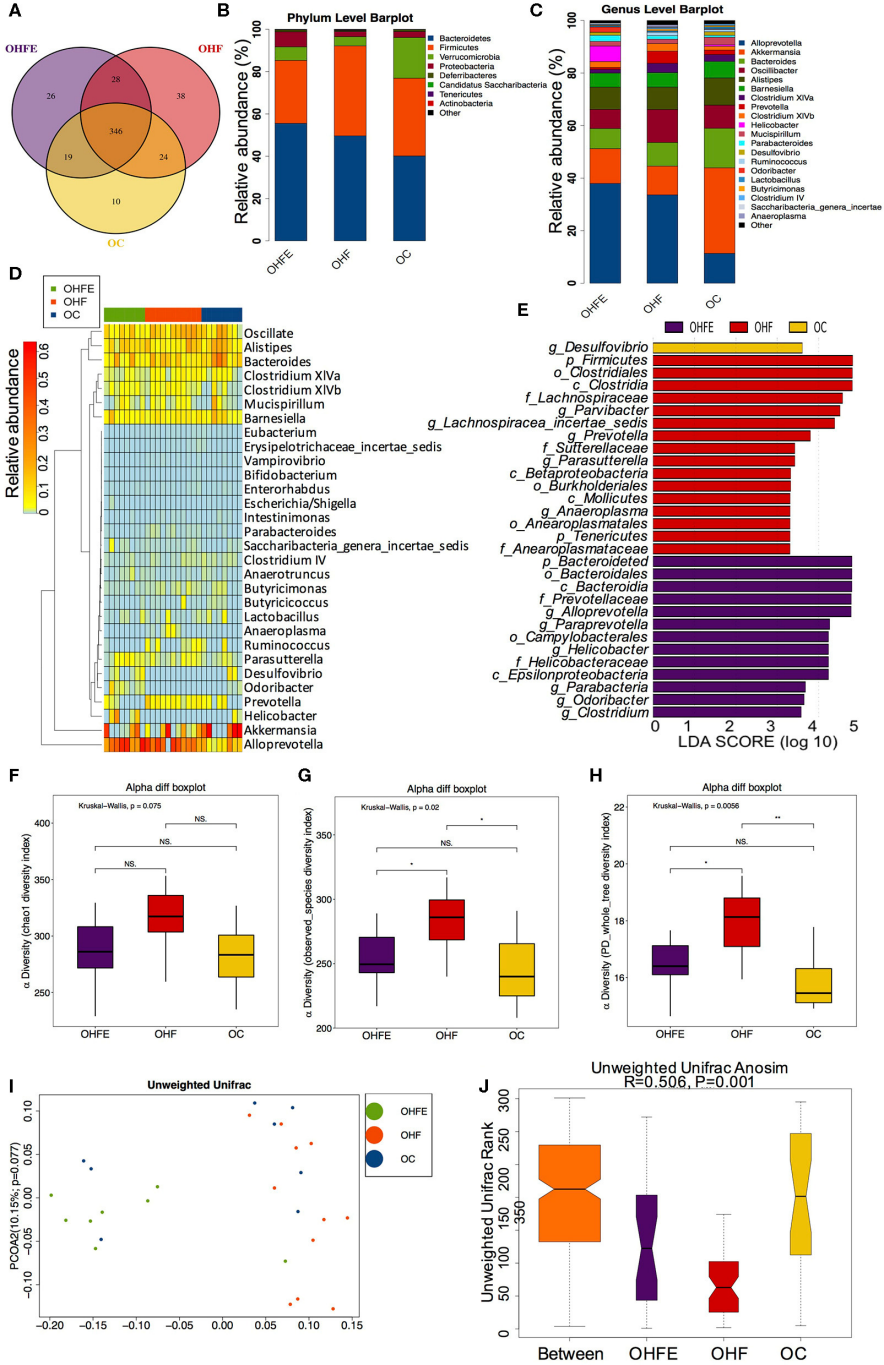
The changes of gut microbiome in male offspring at 24 weeks of age (OC, *n* = 8; OHF, *n* = 11; OHFE, *n* = 8 in each figure). **(A)** Venn diagram of the OUTs; **(B)** Relative abundance of the top 10 germs at the phylum level; **(C)** Relative abundance of the top 20 species at the genus level; Different species analysis among the three groups: **(D)** Heatmap analysis of the different species at the genus level among the three group; and **(E)** LEfSe analysis of the significantly enriched gut microbiota from the phylum level to the genus level; Alpha diversity analysis of the gut microbiome in offspring at 24 weeks: **(F)** chao1; **(G)** observed_species; and **(H)** PD_whole_tree; Beta diversity analysis of gut microbiome: **(I)** PCoA plots of gut microbiome; and **(J)** ANOSIM analysis. OC, offspring of dams fed the normal control diet; OHF, offspring of dams fed the high-fat diet; OHFE, offspring of dams intervened with high-fat diet and exercise. Data are expressed as means ± S.E.M. Mean values show significant differences between the groups: ^*^p < 0.05, ^**^p < 0.01.

In light of the persistent protective effects of maternal exercise on offspring during aging, we further analyzed the gut microbiota in male offspring at 24 weeks of age and explored whether the alterations in dams and 8-week-old male offspring could persist into adulthood. Notably, gut microbiota at 24-week is better separated from maternal gut microbiota than those in offspring at 8 weeks (Supplementary Figure S3). In terms of the gut microbiome in aging male offspring, the Venn diagram showed that there were also large numbers of shared and unique OUTs among the three groups (Figure 6A). And the top abundant phyla were also the same as those in dams and offspring at 8 weeks of age (Figure 6B). Top 20 genera were listed in Figure 6C and significantly different species at the genus level in male offspring at 24 weeks of age were listed in Figure 6D. At the genus level, maternal HFD feeding significantly enriched *Lachnospiracea_incertae_sedis* and *Anaeroplasma* in male offspring. *Anaeroplasma* has been reported to be associated with the development and progression of various chronic metabolic disorders (Robertson et al., 2017). Maternal exercise dramatically increased the abundance of *Helicobacter, Odoricbacter*, and *Clostridium XIVb* in offspring from MHFE group (OHFE) (Figure 6E). Alpha diversity analysis showed that maternal HFD intake significantly increased community richness and diversity compared with mice in the OC group, which was reduced by maternal exercise (Figures 6F–H). And the gut microbiota was well-separated in male offspring at 24 weeks of age among the three groups (ANOSIM *R* = 0.506, *p* = 0.001) (Figures 6I,J). Overall, *Lachnospiracea_incertae_sedis* was commonly abundant in young and aging male offspring from HFD-fed dams, which was decreased by maternal exercise. Additionally, *Odoricbacter* was significantly enriched by maternal exercise in both dams at weaning and offspring at 8 weeks and 24 weeks. These species might play significant roles in mediating the metabolic phenotype induced by a HFD and exercise.

### The Relationship Between Altered Gut Microbiota at the Genus Level and Glucose and Lipid Metabolic Parameters in Dams and Offspring

**Figure 7:**
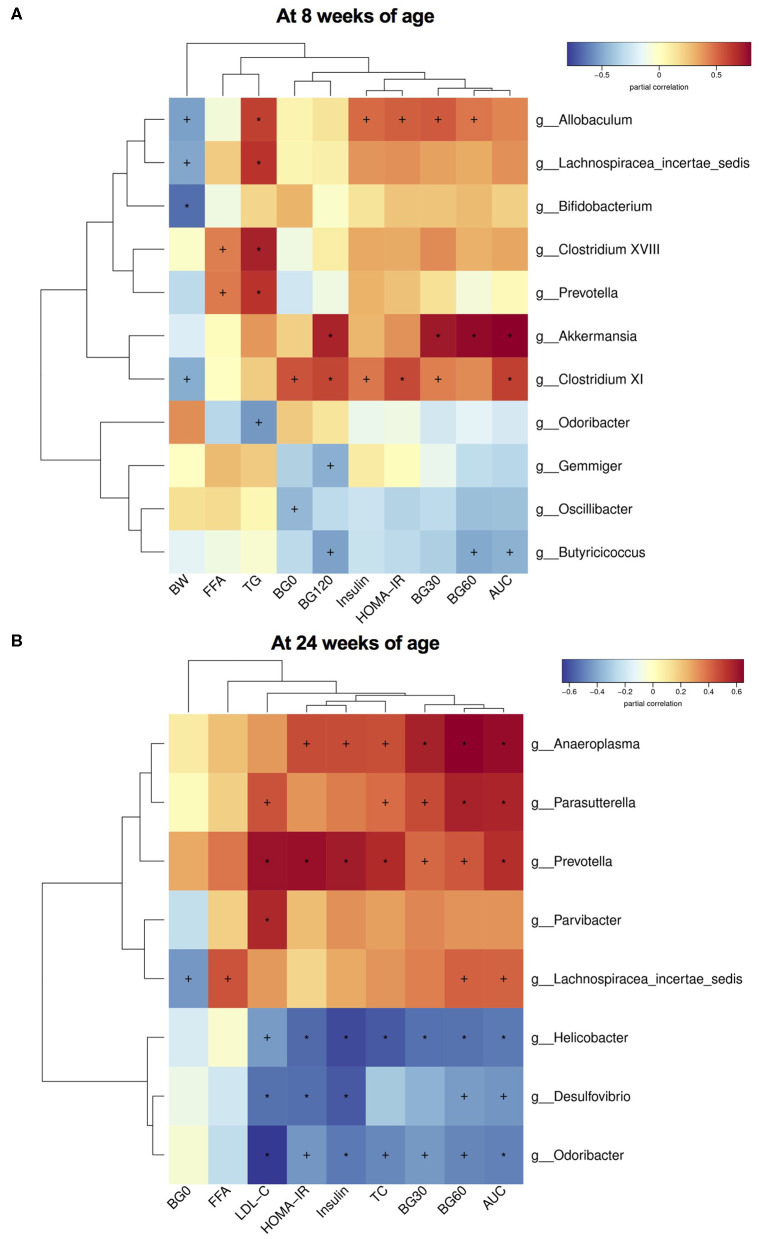
Heatmap of correlation analysis between the changed species at the genus level and glucose and lipid metabolic parameters in male offspring. **(A)** Correlation results in offspring at 8 weeks of age; and **(B)** Correlation results in offspring at 24 weeks of age. BW, body weight; BG0, blood glucose level at 0 min of GTT; BG30, blood glucose level at 30 min of GTT; BG60, blood glucose level at 60 min of GTT; BG120, blood glucose level at 120 min of GTT; AUC, area under the curve of GTT; HOMA-IR, the homeostasis model assessment of insulin resistance; TC, total cholesterol; TG, triglyceride; LDL-C, low-density lipoprotein cholesterol; and FFA, free fatty-acid. The statistics were analyzed by spearman correlation analysis. Values show significant correlation between the genera and metabolic parameters: +*p* < 0.05; ^*^p < 0.01.

Finally, to investigate the relationship between gut microbiota and glucose and lipid metabolism, we performed the correlation analysis between the relative abundance of altered germs at the genus level and metabolic parameters in dams and male offspring. As shown in Table 1, the significantly enriched *Parasutterella, Bifidobacterium, Lactococcus* and *Intestinimonas* in the MC group were all negatively correlated with the blood glucose levels during IPGTT and AUC, among which *Parasutterella, Bifidobacterium*, and *Intestinimonas* were all negatively correlated with the serum LDL-C and TC levels in dams at weaning. As for male offspring at 8 weeks of age, the relative abundance of *Allobaculum* and *Lachnospiracea_incertae_sedis*, which were elevated by maternal HFD, were positively correlated with the serum TG levels. Additionally, the abundance of *Allobaculum* was also positively correlated with the BG30 and BG60 during IPGTT, fasting insulin levels, and HOMA-IR index. In concordance with dams, the significantly enriched *Bifidobacterium* in the C group was negatively correlated with body weight in male offspring. The relative abundance of *Butyricicoccus* which was enriched in offspring of control diet-fed dams was negatively correlated with BG60 and BG120 during IPGTT and AUC. The elevated *Odoricbacter* by maternal exercise was inversely related to the serum TG levels in male offspring at 8 weeks of age (Figure 7A). When it comes to the male offspring at 24 weeks of age, the significantly elevated *Lachnospiracea_incertae_sedis* and *Anaeroplasma* by 9 weeks of maternal HFD were both positively correlated with the BG60 during IPGTT and AUC. Moreover, the relative abundance of *Lachnospiracea_incertae_sedis* was also positively related to the serum FFA levels, and the abundance of *Anaeroplasma* was significantly related to the fasting insulin levels, HOMA-IR, and serum TC levels. By contrast, the significantly elevated *Helicobacter* by maternal exercise was dramatically negatively correlated with BG30, BG60 during IPGTT, AUC, fasting insulin levels, HOMA-IR, serum LDL-C, and TC levels in adult offspring. In particular, the significantly enriched *Odoricbacter* by maternal exercise in dams and male offspring at 8 weeks of age was negatively correlated with not only the BG levels during IPGTT, AUC, insulin levels, and HOMA-IR, but the serum LDL-C and TC levels in adult male offspring (Figure 7B).

**Table 1:** Correlation analysis between the relative abundance of the changed microbial genera and glucose and lipid metabolic parameters in dams at weaning.

**Genera**	**Metabolic parameters**	***p*-value**	***r***
*Parasutterella*	Body weight	0.022	−0.253
	LDL-C	0.002	−0.572
	TC	0.015	−0.462
*Bifidobacterium*	BG30	<0.001	−0.730
	BG60	<0.001	−0.677
	AUC	0.004	−0.534
	Insulin	0.056	−0.372
	LDL-C	0.022	−0.440
	TC	0.037	−0.403
*Lactococcus*	Body weight	0.038	−0.402
	BG30	<0.001	−0.621
	BG60	0.004	−0.541
	AUC	0.011	−0.483
*Intestinimonas*	Body weight	<0.001	−0.611
	BG60	0.017	−0.456
	AUC	0.006	−0.518
	Insulin	0.026	−0.428
	LDL-C	0.005	−0.522
	TC	0.005	−0.529

*The statistics were analyzed by spearman correlation analysis*.

*LDL-C, low-density lipoprotein cholesterol; TC, total cholesterol; BG30, blood glucose level at 30 min of GTT; BG60, blood glucose level at 60 min of GTT; AUC, area under the curve*.

## Discussion

The adverse nutritional environment in early life might be one of the important factors in deciphering the rapid development of obesity and metabolic disorders. During the last decade, growing numbers of animal experimental models have demonstrated that maternal obesity or HFD resulted in increased offspring susceptibility to metabolic disorders (Campodonico-Burnett et al., 2020; Costa et al., 2020). Concordantly, our study also demonstrated that maternal HFD not only led to glucose and lipid metabolic disturbances in dams but also programmed glucose intolerance and disorders of serum lipid profiles in male offspring from young into adulthood.

Therefore, early life might be a critical window for cutting off the intergenerational transmission of metabolic diseases. In light of the multiple metabolic benefits of regular exercise, we gave the mothers a voluntary wheel running training 3 weeks before mating and during pregnancy in this study to determine whether maternal exercise can prevent the harmful impacts of maternal HFD on dams and offspring. The experimental results were consistent with the previous studies in support of the intergenerational health benefits of maternal exercise (Stanford et al., 2015, 2017; Zheng et al., 2020). Indeed, maternal exercise significantly mitigated the deleterious effects of the HFD on metabolism, including insulin resistance in dams at weaning and glucose and lipid metabolic disorders in male offspring from young into adult. These data indicated that maternal exercise was critical for improving metabolic health in both dams and offspring.

There might be numerous changes responsible for the improved metabolic phenotypes in both dams and offspring after maternal exercise. Given the pivotal role of gut microbiota plays in metabolic health and the confirmed link of exercise with the intestinal microbial community (Liu et al., 2020; Motiani et al., 2020), we discussed the potential contributions of gut microbiota to the intergenerational benefits of maternal exercise. Our data demonstrated that maternal exercise significantly modified gut microbiota in dams fed HFD at weaning, including both structure and composition, which was inconsistent with the results of another recent study. The Sprague-Dawley rats were fed with an obesogenic diet and were given access to a running wheel 10 days before pregnancy until delivery in Bhagavata Srinivasan's study. The obesogenic diet included two types of the high-fat pellet (SF03-020: 43% fat, 16% protein, and 41% carbohydrate by energy, and SF03-002: 59% fat, 14% protein, and 27% carbohydrate by energy; Specialty feeds, Australia) supplemented with one of three different western foods (selected from cakes, potato chips, biscuits, meat pie, pasta with lard, and oats mixed with condensed milk). They did not observe marked impacts of maternal exercise on gut microbiota in dams fed the obesogenic diet (Bhagavata Srinivasan et al., 2018). Nevertheless, in this study, we fed mice with a high-fat diet of D12492 (Research diet) with 60% of the calories as fat, 20% as carbohydrate, and 20% as protein. Additionally, we used the C57BL/6 inbred mouse model other than outbred SD rats. Both of dietary composition and animal strain, as well as exercise paradigms and time, have been reported to be the potential influencing factors for the gut microbiota, which might explain the different results between the studies (Harris et al., 2018). Given the significantly altered microbiome observed in both mothers and offspring in this study, we further explored whether these species were associated with or even can explain the improved metabolic phenotypes.

Maternal exercise 3 weeks before mating and during pregnancy markedly elevated the abundance of *Alloprevotella* at weaning. *Alloprevotella* is a Gram-negative germ and is weakly to moderately saccharolytic. It has been confirmed that *Alloprevotella* can produce acetic and succinic acids as end products of fermentation (Downes et al., 2013). A recent study provided evidence that the improved lipid profiles, including a reduction in TG, TC, LDL-C, and hepatic lipid accumulation, induced by polyunsaturated fatty acids supplementation in HFD-fed mice were related with the elevated abundance of *Alloprevotella* (Li et al., 2019b). The enrichment of *Alloprevotella* has also been shown to be associated with the systemic metabolic benefits of the intake of functional Oligosaccharides (Cheng et al., 2017), lactulose (Zhang et al., 2019), probiotics (Kong et al., 2019), and berberine organic acid salt (Cui et al., 2018) in animal models. In addition, our previous study demonstrated that maternal genistein intake also elevated the relative abundance of *Alloprevotella* in adult male offspring, which might play important roles in the systemic metabolic improvement (Zhou et al., 2019a). In contrast, its abundance was negatively correlated with obesity, hyperglycemia, and insulin resistance (Shang et al., 2017). Stimulating gastrointestinal motility to protect the gut mucosal barrier and regulating energy metabolism and insulin resistance via indirectly producing short-chain fatty acids (SCFAs) were reported to be the potential actions of *Alloprevotella*. Consistent with these findings, our data showed the enriched abundance of *Alloprevotella* in the exercise group. This might play a role in deciphering the benefits of exercise on the mothers.

Moreover, *Barnesiella* was also elevated by maternal exercise in dams. As the most abundant novel germ in healthy human gut microbiota (Wei et al., 2018), *Barnesiella* is a member of the family *Porphyromonadaceae* from the phylum Bacteroidetes, which can mainly produce butyric and iso-butyric acids other than small numbers of succinic, propionic and acetic acids (Sakamoto et al., 2009). Numerous studies have supported the relationship between the increase in the abundance of *Barnesiella* and improved hyperlipidemia, glucose intolerance, hyperinsulinemia, and hepatic steatosis (Anhe et al., 2017; Henning et al., 2018; Li et al., 2018b, 2019a; Wei et al., 2018; Perez-Burillo et al., 2019). Additionally, *Barnesiella* also played some roles in protecting against the dextran sulfate sodium (DSS)-induced colitis (Li et al., 2018a) and infection of *Clostridium diffcile* (Milani et al., 2016). Therefore, the reduction of susceptibility to inflammation might play an important role in the metabolic benefits of *Barnesiella* (Li et al., 2018a; Wei et al., 2018; Ye et al., 2019). To our knowledge, this study showed the enrichment of *Barnesiella* in mothers at weaning by exercise during the special period-3 weeks before mating and during pregnancy for the first time, which prompted us to do further microbiota transplantation experiments to validate the importance of this germ to metabolism.

When it comes to offspring, we also observed great alterations in the gut microbiota among three groups, although they were all fed standard chow diet and stayed sedentary after weaning. Interestingly, maternal HFD feeding significantly enriched the genus *Lachnospiracea_incertae_sedis* in male offspring at both 8 weeks and 24 weeks. *Lachnospiracea_incertae_sedis* is a member of *Lachnospiraceae*. A clinical trial has observed that *Lachnospiracea_incertae_sedis* is markedly enriched in patients with non-alcoholic fatty liver disease (NAFLD) and can discriminate the patients from healthy controls (Shen et al., 2017). In terms of animal model experiments, Zeng et al. showed that chronic HFD not only increased plasma leptin, interleukin 6, and tumor necrosis factor α levels but enriched *Lachnospiraceae* in mice intestine (Zeng et al., 2016). More significantly, another study transplanted a *Lachnospiraceae* strain to germ-free *ob/ob* mice and observed significantly elevated fasting blood glucose levels as well as liver weight and fat mass, and decreased plasma insulin levels and HOMA-β in colonized mice compared with germ-free mice. They exhibited evidence for *Lachnospiraceae* as a contributor to the development of obesity and diabetes in mice (Kameyama and Itoh, 2014). Although the function of *Lachnospiracea_incertae_sedis* has not been clearly clarified, our research added more evidence for the positive relationship between the systemic glucose and lipid metabolic parameters and the abundance of this species. Maternal exercise attenuated the deleterious effects of HFD on offspring metabolism accompanied by loss of this unfavorable bacteria from young into aging. Therefore, deficiency of this harmful germ in offspring from exercise mothers might be a potential mediator for the intergenerational effects.

More interestingly, our data demonstrated that maternal exercise consistently enriched the genus *Odoribacter* in both dams and male offspring, which was negatively related to the glucose and lipid metabolic parameters. Although it is largely unclear whether this bacterium was transmitted across generations or it was programmed by other factors, our study highlighted the extremely important role of this species in mediating the metabolic benefits of exercise. As a member of family *Porphyromonadaceae, Odoribacter* is an anaerobic Gram-negative genus and a known SCFA producer (Goker et al., 2011; Vital et al., 2014). An animal experiment transplanted fecal microbiota from exercise donors to HFD-fed mice and observed that the transmissible beneficial effects of fecal microbiota transplantation (FMT) were associated with genus *Odoribacter*, which might contribute to the reduced inflammation (Lai et al., 2018). Additionally, a clinical trial showed an inverse correlation between the abundance of *Odoribacter* and systolic blood pressure in overweight and obese women (Gomez-Arango et al., 2016). The metabolic improvements of multiple prebiotics and probiotics were also indicated to be associated with the elevated abundance of *Odoribacter*, accompanied by increased SCFAs levels (Cheng et al., 2018; Dai et al., 2019; Perez-Burillo et al., 2019). For the first time to our knowledge, our study indicated that the enrichment of genus *Odoribacter* in both dams and offspring might be one of the critical factors for the intergenerational beneficial impacts of maternal exercise.

Overall, this study identified a group of species-SCFAs producers, such as *Intestinimonas, Butyricicoccus, Alloprevotella, Barnesiella*, and *Odoribacter*, which are enriched at control groups and exercise intervention groups but were decreased in HFD groups. SCFAs mainly include acetate, propionate, butyrate, and iso-butyrate, which are produced by intestinal microbial fermentation of dietary fiber and complex carbohydrates. Most of the SCFAs producers play important roles in the host health. Evidence has shown that butyrate downregulates pro-inflammatory cytokines by activating G-protein coupled receptors (GPRs), inhibiting histone deacetylases, and regulating colonic regulatory T cells (McNabney and Henagan, 2017). Moreover, butyrate was able to pass through the enterocytes and reach and influence the peripheral tissues, which acts as a mediator for the crosstalk between the gut microbiome and other tissues and the regulation of systemic metabolism (den Besten et al., 2013). This is the first study to highlight the importance of SCFAs-producing species in mediating the intergenerational effects of maternal exercise. In terms of the potential way of these SCFAs producers to regulate the intergenerational effects, a recent study determined that SCFAs such as acetate, propionate, and butyrate from maternal gut microbiota during pregnancy can be sensed by their receptors GPR41 and GPR43 in the embryonic intestinal tract and pancreas, which impacted the development of the metabolic system and programmed offspring resistance to obesity. Consistently, maternal high-fiber diet feeding and a SCFA supplementation in this study also reduced offspring susceptibility to obesity and metabolic disorders induced by HFD (Kimura et al., 2020). This made it possible that maternal exercise might also protect offspring metabolism via SCFAs. However, the levels of SCFAs and the specific mechanism by which explains the metabolic benefits of SCFAs-producing bacteria is unclear in this study, which needs out further exploration.

In conclusion, maternal 9 weeks of HFD feeding led to glucose and lipid metabolic disorders in both dams and male offspring. However, maternal exercise before and during pregnancy significantly improved the metabolic disturbances induced by HFD in both mothers and offspring from young into adulthood. For the first time to out knowledge, this study showed that maternal exercise resulted in persistent changes in gut microbiota from dams to adult offspring. The reduction in unfavorable bacteria such as *Lachnospiracea_incertae_sedis* and the marked enrichment of SCFAs producers, especially the *Odoribacter*, might be the critical contributor to the intergenerational metabolic benefits of maternal exercise. There are still several limitations in our research. First, we only explore the effects of maternal exercise on male offspring. Female offspring requests for further exploration. Besides, we need to detect the level of SCFAs in the future to confirm the concrete impacts of SCFAs producers. Last, we still have no idea about the causal relationship between maternal exercise, altered microbiota and metabolic parameters. More experiments about FMT and antibiotic intervention are required to investigate the exact association.

## Data Availability Statement

The datasets generated for this study can be found in the Sequence Read Archive (SRA) database (PRJNA573981).

## Ethics Statement

The animal study was reviewed and approved by The institutional animal care and use committee of the Peking Union Medical College Hospital (Beijing, China, SYXC-2014-0029).

## Author Contributions

XX and JZ designed the experiments. LZ and MD performed the experiments. LZ analyzed the data and drafted the manuscript. XX, ML, QZ, and MY reviewed the manuscript. All authors approved the submitted version of the manuscript.

## Conflict of Interest

The authors declare that the research was conducted in the absence of any commercial or financial relationships that could be construed as a potential conflict of interest.
