# Supplementary material for: Maternal Exercise Improves High-Fat Diet-Induced Metabolic Abnormalities and Gut Microbiota Profiles in Mouse Dams and Offspring
Source: Front Cell Infect Microbiol. 2020 Jun 17;10:292. doi: 10.3389/fcimb.2020.00292 (PMC7311581; doi:10.3389/fcimb.2020.00292)
Supplement: Supplementary file 1 [file Data_Sheet_1.DOCX]

Supplementary Material

**Table S1.** The nutritional compositions of two types of diet. CD, normal control diet; HFD, high-fat diet.

| **Ingredients** | **CD(g)** | **HFD(g)** |
| --- | --- | --- |
| Casein | 200 | 200 |
| L-Cystine | 3 | 3 |
| Corn Starch | 506.2 | 0 |
| Maltodextrin 10 | 125 | 125 |
| Sucrose | 68.8 | 68.8 |
| Cellulose, BW200 | 50 | 50 |
| Soybean Oil | 25 | 25 |
| Mineral Mix S10026 | 10 | 10 |
| Vitamin Mix, V10001 V10001 | 10 | 10 |
| Choline Bitartrate | 2 | 2 |
| Lard | 20 | 245 |
| DiCalcium Phosphate | 13 | 13 |
| Calcium Carbonate | 5.5 | 5.5 |
| Potassium Citrate, 1 H2O | 16.5 | 16.5 |
| FD&C Blue Dye #1 | 0.01 | 0.05 |
| FD&C Yellow Dye #5 | 0.04 | 0 |
| Total | 1055.05 | 773.85 |

**Figure S1.** Alpha diversity of gut microbiota in dams at weaning (MC, n=8; MHF, n=11; MHFE, n=8 in each figure). (A) Chao1 index; (B) Obeserved_species; and (C) PD_whole_tree. MC, dams fed the normal control diet; MHF, dams fed the high-fat diet; MHFE, dams intervened with a high-fat diet and exercise.

**Figure S2.** Alpha diversity of gut microbiota in male offspring at 8 weeks of age (C, n=6; HF, n=9; HFE, n=6). (A) Chao1 index; (B) Obeserved_species; and (C) PD_whole_tree. C, offspring of dams fed the normal control diet fed; HF, offspring of dams fed the high-fat diet; HFE, offspring of dams intervened with a high-fat diet and exercise.

**Figure S3.** PCA plot of OTUs among the nine groups in both dams and offspring (MC, n=8; MHF, n=11; MHFE, n=8; C, n=6; HF, n=9; HFE, n=6; OC, n=8; OHF, n=11; OHFE, n=8). MC, dams fed the normal control diet; MHF, dams fed the high-fat diet; MHFE, dams intervened with a high-fat diet and exercise; C, offspring of dams fed the normal control diet fed at 8 weeks; HF, offspring of dams fed the high-fat diet at 8 weeks; HFE, offspring of dams intervened with a high-fat diet and exercise at 8 weeks; OC, offspring of dams fed the normal control diet at 24 weeks; OHF, offspring of dams fed the high-fat diet at 24 weeks; OHFE, offspring of dams intervened with a high-fat diet and exercise at 24 weeks.

**
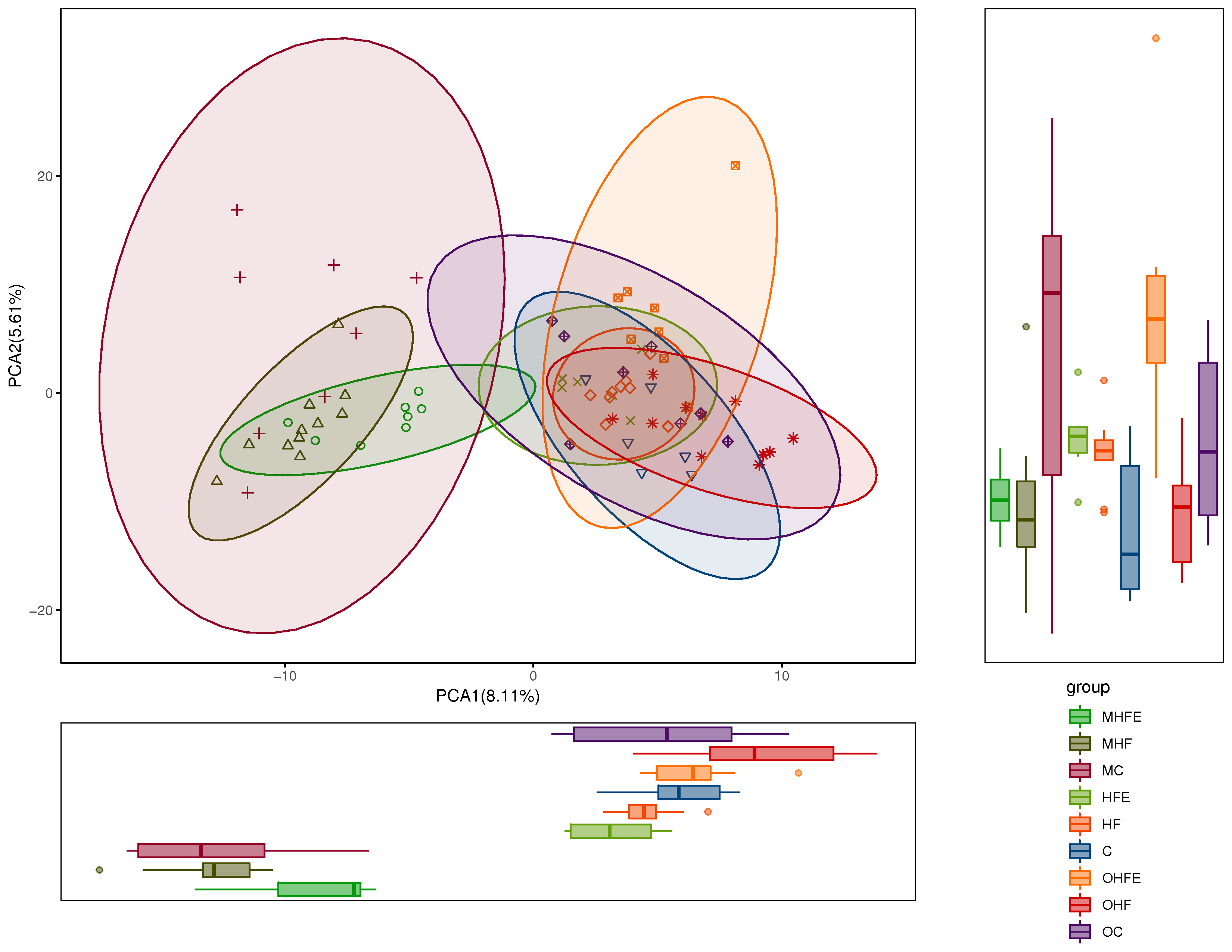
**

**
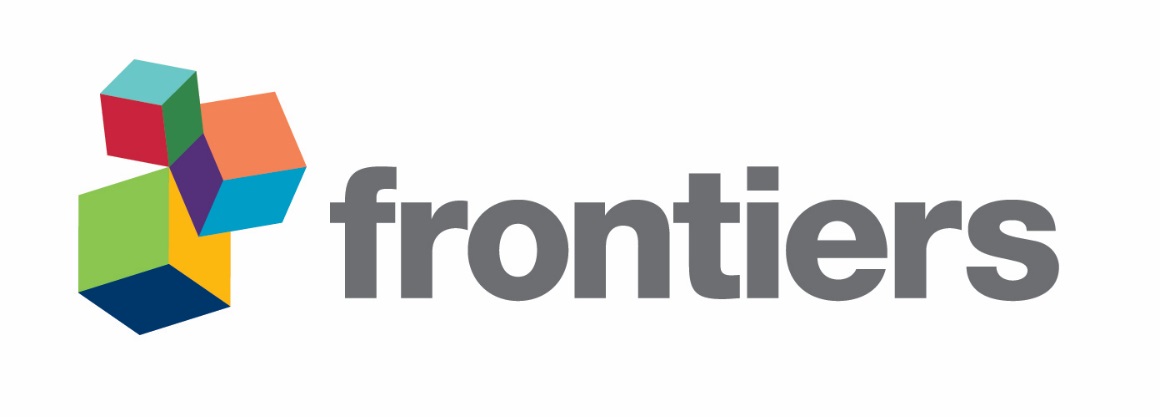
**
